# The interplay between ego-resiliency, math anxiety and working memory in math achievement

**DOI:** 10.1007/s00426-024-01995-0

**Published:** 2024-06-28

**Authors:** Eleonora Doz, Alessandro Cuder, Sandra Pellizzoni, Federica Granello, Maria Chiara Passolunghi

**Affiliations:** Department of Life Sciences, University of Trieste, via Weiss 21 (Building W), Trieste, 34128 Italy

**Keywords:** Ego-resiliency, Math anxiety, Working memory, Math performance, Word problems, Primary school

## Abstract

Previous research has suggested that math anxiety may contribute to poor math performance by interfering with working memory. However, only a limited number of studies investigated the mediating role of working memory in the math anxiety-math performance link in school-aged children. Unlike math anxiety, ego-resiliency is a personality resource that promotes the management of challenges and has been positively associated with math performance and negatively with anxiety. Nevertheless, there is still limited understanding regarding the specific role of ego-resiliency in math learning and how it relates to math anxiety. This study aimed to investigate conjunctly the interplay between primary school children’s ego-resiliency, math anxiety, working memory, and performance on two different math tasks (i.e., arithmetic task and word problem-solving task), after controlling for general anxiety and age. The study involved 185 Italian children from grades 3 to 5. Serial multi-mediational analyses revealed that: (1) ego-resiliency has a positive indirect effect on math achievement through two paths - math anxiety, and math anxiety and working memory; (2) the study replicated previous findings showing that working memory partially mediated the relationship between math anxiety and math performance; (3) similar patterns of results were found for both math skills. The study identifies ego-resiliency as a possible protective factor in the development of math anxiety and suggests that ego-resiliency could be worth considering when designing interventions aimed at reducing negative emotions towards mathematics.

## Introduction

Mathematical skills represent one of the key competencies necessary for lifelong learning (European Parliament and the Council of European Union, 2006; Holmlund et al., 2018). Having adequate mathematical abilities is necessary for a variety of academic and ordinary life situations, ranging from school examinations to managing personal finances and making career choices (Ansari et al., 2012; Kucian & von Aster, 2015). Yet, many students from primary school onwards manifest specific difficulties in this academic domain (Organization for Economic Cooperation and Development [OECD], 2013). Therefore, in order to foster a productive learning environment, it is essential to elucidate how different factors may influence learners’ math performance.

Previous research has extensively investigated the deleterious effects of negative emotions, namely math anxiety (MA), on students’ math achievement (Dowker et al., 2016). Studies conducted on adults indicated that MA would interfere with cognitive resources (i.e., working memory–WM) leading to poor math achievement (Eysenck & Calvo, 1992; Skagerlund et al., 2019). On the other hand, personal protective factors such as ego-resiliency (ER) have been found to support and sustain children’s math learning (e.g., Donolato et al., 2019, 2020). In particular, ego-resilient pupils would be better at adapting to stressful situations and managing emotions and worries, exhibiting therefore lower anxiety levels (Mammarella et al., 2018; Putwain et al., 2013). However, research has yet to consider how the interplay between ER, MA and WM influences math performance concurrently, especially in school-aged children who are in the process of learning mathematics. Moreover, it must be noted that the effects of the abovementioned variables were vastly explored in relation to simpler arithmetic skills (such as calculation skills; see Cuder et al., 2023; Korem et al., 2022; Skagerlund et al., 2019; Soltanlou et al., 2019). Little is known about how ER, MA and WM may differentially contribute to individual differences in proficiency in word problem-solving, which is defined as the ability to solve math problems presented in a short narrative notation rather than in a numerical notation (Verschaffel et al., 2000). Indeed, the role of emotional, cognitive and temperamental factors for this kind of task, where the problem must be translated from a linguistic to a mathematical structure, are likely to be different from those invoked in basic arithmetic and procedural skills (Doz et al., 2023; Wu et al., 2017; Zhang et al., 2019). To fill these gaps, the present study aims to examine conjunctly the interrelated contributions of affective (i.e., MA), cognitive (i.e., WM) and temperamental (i.e., ER) factors on two different aspects of mathematics (i.e., arithmetic skills and word problem-solving) in primary school children. In the next paragraphs, we review relevant literature on the effect of MA on math achievement, the mediational role of WM in the MA-math performance link and, finally, the relationship between ER and mathematics.

### Math anxiety and math performance

As mentioned above, one of the most extensively studied emotional factors in math learning is MA. MA is commonly defined as a specific feeling of tension and anxiety that hinders the manipulation of numerical stimuli in everyday life and academic situations (Richardson & Suinn, 1972). While MA correlates with other forms of anxiety, such as general anxiety (Ashcraft & Moore, 2009; Donolato et al., 2020; Hart & Ganley, 2019) and test anxiety (Caviola et al., 2022; Cipora et al., 2022; Devine et al., 2012), it is regarded as a distinct and specific construct (Kazelskis et al., 2000; Mammarella et al., 2018).

A substantial body of research demonstrated the detrimental impact of MA on math achievement: highly math-anxious individuals tend to make more errors on math tasks and/or take longer to respond than individuals with low MA (for reviews, see Barroso et al., 2021; Caviola et al., 2022). Notably, some studies suggest the existence of a negative correlation between MA and math attainments as early as in primary school children (e.g., Ramirez et al., 2013; Sorvo et al., 2019; Szczygieł, 2021). This is a significant concern since the development of MA in early school years can lead to avoidance of future math-intensive activities and make the acquisition of mathematical skills more difficult, further exacerbating negative emotions (Ashcraft, 2002; Szczygieł, 2020). Thus, more research is needed to elucidate the role and mechanisms of MA, specifically in primary school students.

### Math anxiety, working memory and math performance

Although the influence of MA on math performance has been consistently demonstrated, the mechanisms underlying this relationship are not completely clear. Thus far, WM has been the most studied factor that could account for how MA may influence math performance (Ashcraft & Kirk, 2001; Justicia-Galiano et al., 2017). WM is defined as a limited capacity cognitive system that allows individuals to hold and simultaneously manipulate information over brief periods of time (Baddeley, 1986; Baddeley & Hitch, 1974). According to the classical multicomponent model (Baddeley, 2000; Baddeley & Hitch, 1974), WM is distinguished by: (1) verbal WM subsystem, which is responsible for the temporary storage of verbal information, such as words and numbers; (2) visuo-spatial WM subsystem, which allows the temporary storage of visual and spatial information, such as colours, figures and positions in space; and (3) the central executive, which is involved in regulating, manipulating, and processing the stored verbal and visuo-spatial information. More recently, Baddeley (2000) added to the model the episodic buffer, which is characterized by multidimensional storage that forms an interface between the subsystems of WM, long-term memory and the central executive. However, the episodic buffer is less studied in developmental samples (Wang et al., 2015).

WM plays a pivotal role in math achievement (for a review, see Peng et al., 2016), since solving math tasks requires applying multiple procedures, storing intermediate computational results and/or numerical information, as well as remembering task rules. Recent research suggests that visuo-spatial WM is particularly relevant to math learning, while verbal WM is more closely associated with reading attainment (Giofrè et al., 2018). Moreover, during primary school years, visuo-spatial WM becomes especially important for tackling new and complex math tasks (e.g., Ashkenazi et al., 2013; Li & Geary, 2013; Szűcs et al., 2014), serving as a reliable predictor of math performance (Allen & Giofrè, 2021; Liang et al., 2022). Given the above-mentioned evidence, in our study we decided to focus on the visuo-spatial WM component.

According to the Processing Efficiency Theory proposed by Eysenck and Calvo (1992), emotional and cognitive factors would interact. In particular, anxiety would generate worries and intrusive thoughts, which may overload WM resources and, consequently, disrupt concurrent task performance (Ashcraft & Krause, 2007). Therefore, WM would mediate, at least partly, the MA-math performance link. Studies involving adult population mainly confirm the Processing Efficiency Theory and the mediating role of WM (e.g., Ashcraft & Kirk, 2001; DeCaro et al., 2010; Ganley & Vasilyeva, 2014; Miller & Bichsel, 2004; Skagerlund et al., 2019). Support for this theory is also provided by recent meta-analytic findings that showed a negative correlation between MA and WM capacity (*r* = − .168) and found that WM mediates the relationship between MA and math performance with a mean effect size of − 0.092 (Finell et al., 2022). However, research on school-aged children remains relatively limited (Justicia-Galiano et al., 2017; Pellizzoni et al., 2022; Szczygieł, 2021). For instance, Živković et al. (2023) investigated the mediating role of WM and self-competence in the relationship between MA and arithmetic reasoning in students from third, fifth and seventh grade. They found that MA affected the visuo-spatial WM component, which subsequently influenced students’ math performance. Similar results were obtained by Soltanlou et al. (2019), who examined the relationship between MA, WM and multiplication learning in fifth graders using an intervention paradigm. Nevertheless, it must be noted that the majority of these studies on school-aged children (i.e., Soltanlou et al., 2019; Szczygieł, 2021; Živković et al., 2023) did not control for children’s general anxiety, which was observed to be related to MA sharing a moderate amount of variance (see Donolato et al., 2020; Hembree, 1990; Luttenberger et al., 2018). Understanding the unique impact of MA on WM in primary school children is crucial from both theoretical and practical perspectives. Therefore, more studies investigating this relationship in developmental samples, while considering students’ general anxiety, is needed.

### Ego-resiliency and math performance

In contrast to MA – which disrupts math performance – children possess several personal assets that sustain positive outcomes in their personal, social, and academic life domains (Eisenberg et al., 1997; Masten, 2001). Among these, ER is considered to play a key role (Donolato et al., 2020). ER refers to a temperamental or personality resource that allows individuals to be flexible and resourceful in adapting to external and internal stressors and recover quickly from day-to-day adversities (Block & Block, 1980; Caspi & Silva, 1995; Fletcher & Sarkar, 2013). Thus, resilient people are able to maintain or rapidly return to their prior level of functioning after confronting a stressful situation (Block & Block, 2006) and therefore avoid negative (emotional, motivational or cognitive) consequences.

In school environments, students frequently experience some level of challenge, adversity or pressure, such as evaluative stress, scarce performance, demanding tasks, and difficulties in understanding concepts. This could be particularly true for mathematics, which is considered a complex school subject, and often reported as one of the most demanding, stressful and strenuous in the school curriculum (Ashcraft et al., 2007; Ashcraft & Ridley, 2005). According to the definition given above, ER could be a relevant personal resource for managing school challenges and emotional difficulties during math learning (Alessandri et al., 2017). In this respect, several studies reported a positive association between students’ ER and math achievement supporting the conviction that amongst children of similar math ability, ego-resilient individuals perform better on challenging math tasks (Alessandri et al., 2017; Donolato et al., 2019, 2020; Kwok et al., 2007; Putwain et al., 2013).

According to the theoretical conceptualization of ER proposed by Block and Block (1980, 1996), ego-resilient individuals experience lower levels of anxiety or other negative affects since they are able to maintain a sufficient adaptational system and are better at emotional regulation (Eisenberg et al., 2011; Lee & Johnston-Wilder, 2017; Martin & Marsh, 2006). Supporting this theoretical perspective, previous research showed a negative association between ER and general anxiety (Block & Gjerde, 1990; Chuang et al., 2006; Donolato et al., 2020; Huey & Weisz, 1997). For instance, Mammarella et al. (2018) employing a latent profile analysis found that primary school children with a low anxiety risk profile (general anxiety, test anxiety and math anxiety) scored higher on resilience than those at higher risk for anxiety. These findings suggest that ER could act as a protective factor in the development of various forms of anxiety. Accordingly, Putwain et al. (2013) examined whether test anxiety mediated the relationship between ER and test performance (in math and English) in primary school children, after controlling for their prior abilities. The authors found a significant indirect effect of resilience on test performance through test anxiety. That is, students with high ER experienced lower test anxiety and therefore performed better than their less resilient counterparts who experienced greater test anxiety. A similar mediating mechanism could be hypothesized with other specific forms of anxiety, such as MA: ER might positively impact math achievement by boosting students’ ability to manage MA (Lee & Johnston-Wilder, 2017). However, to the best of our knowledge no study has examined the mediating role of MA in the ER-math performance relationship in primary school children.

### Present study

**Fig. 1:**
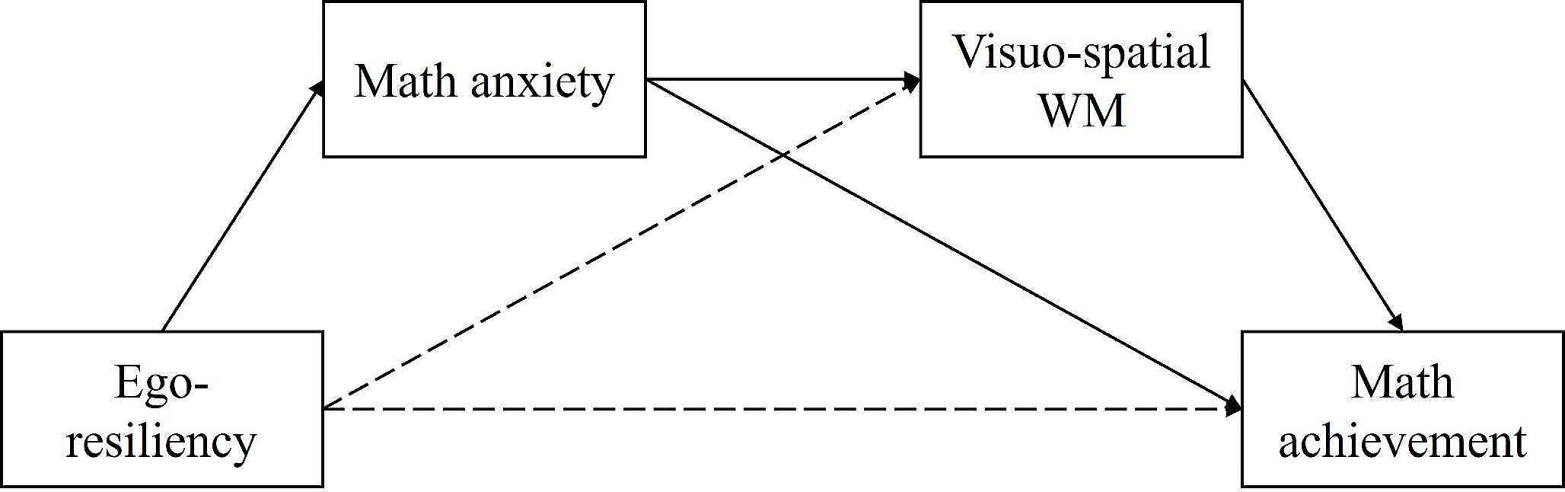
Illustration of the hypothesized theory-based serial multi-mediational model Note: The model assumes indirect effects from ego-resiliency to math achievement through (1) math anxiety and (2) math anxiety and WM. The dashed lines represent hypothesized non-significant relations

Considering the scarce findings on the topic, the main aim of this study was to investigate the simultaneous role of ER, MA and visuo-spatial WM on primary school children’s math achievement on two different tasks (i.e., arithmetic skills and word problem-solving), while controlling for general anxiety and age. In particular, we sought to test a theory-based serial multi-mediational model (see Fig. 1) and verify whether the relationship between ER and math performance would be serially mediated by MA (first mediator) and visuo-spatial WM (second mediator). Involving multiple mediators could better explain how the interplay between different variables influence students’ math attainments (Hayes, 2013; Justicia-Galiano et al., 2017).

Based on Block & Block’s (1980) theory and more recent evidence that demonstrated that ego-resilient children show greater emotional management and lower anxiety levels (e.g., Donolato et al., 2019; Mammarella et al., 2018; Putwain et al., 2013), we expected ER to be negatively related to MA. MA would then have both a direct (Pellizzoni et al., 2022; Skagerlund et al., 2019; Wu et al., 2017) and an indirect negative effect through visuo-spatial WM on math performance (Finell et al., 2022; Živković et al., 2023).

As previously mentioned, two different types of math skills were included as dependent variables given that the majority of existing studies focused on simple arithmetic tasks (Commodari & La Rosa, 2021; Soltanlou et al., 2019; Živković et al., 2023), neglecting other more complex and demanding math abilities such as word problem-solving (Doz et al., 2023; Gilmore, 2023; Passolunghi et al., 2019; Wu et al., 2017). In order to investigate the possible different contribution of temperamental, emotional and cognitive factors on different tasks, our study aimed to further previous research by taking into consideration both math skills, i.e. arithmetic skills and word problem-solving.

### Method

#### Participants

A total of 221 third, fourth and fifth-grade children attending five public primary schools located in northeast Italy were enrolled in the study. Only typically developing children were considered for the subsequent analysis. Therefore, 19 participants with special educational needs, intellectual disability or neurological or genetic disorders were excluded from the sample. Six participants were excluded from the analysis due to missing data (they were absent on at least one of the testing sessions). One participant produced outlier scores on math anxiety measure and was handled with listwise deletion and removed from the analysis. Thus, the final sample analysed in this study consisted of 185 children (102 girls, 83 boys) with a mean age of 9.47 years (*SD* = 0.88, min = 8, max = 11). All students were Caucasian, and their socio-economic status was mainly middle class, established on the basis of school records. All the students were native Italian speakers or fluent in Italian. For sample size estimation, an a priori power analysis was conducted using a simulation method in R, version 4.2.3 (R Core Team, 2020). Results indicated that the minimum sample size needed to achieve statistical power of 0.80 for detecting the hypothesized indirect effect, at a significance criterion of α = 0.05, was *N* = 181. Thus, our sample size of *N* = 185 is adequate.

The study received approval by the ethical committee of the University of XXX and was conducted in compliance with the Declaration of Helsinki, the ethical guidelines of the Italian Association of Psychology and the ethical code of the Italian Register of Professional Psychologists. Written informed parental consent was obtained before assessing the students. All children participated voluntarily and received a small gift (i.e., a sticker collection) as an appreciation for their participation.

### Materials

#### Ego-resiliency

Ego-resiliency was measured using the *Ego Resiliency Scale* (ER89; Block & Kremen, 1996; Italian adaptation by Donolato et al., 2019). This is a questionnaire consisting of 14 statements (e.g., “I quickly get over and recover from being startled”). Participants were asked to indicate on a 4-point Likert scale (1 = *does not apply at all*; 4 = *applies very strongly*) the degree to which they agree to each item. The total score could range from 14 to 56, with higher scores indicating greater ER. The reliability of the questionnaire in the present sample was adequate (Cronbach’s α = 0.75).

#### Math anxiety

The *Abbreviated Math Anxiety Scale* (AMAS; Hopko et al., 2003; Italian adaptation for primary school children by Caviola et al., 2017) was used to evaluate children’s level of MA. AMAS is a brief questionnaire that comprises nine statements describing different situations involving mathematical activities (i.e., math learning and math testing). Children had to determine how anxious they would feel in each depicted situation, using a 5-point Likert scale (1 = *a little anxious*; 5 = *extremely anxious*). The total score could range from 9 to 45, with higher scores indicating higher levels of MA. In the present sample, a good reliability of the scale was observed (Cronbach’s α = 0.83).

#### General anxiety

The *Revised Children’s Manifest Anxiety Scale-Second Edition* (RCMAS-2; Reynolds et al., 2012) is a self-report questionnaire used to assess general anxiety in children and adolescents aged 6–19. In the present study, the Short Form of the RCMAS-2 was employed. It consists of 10 items (e.g., “I feel someone will tell me I do things the wrong way”) to which children must respond with a yes/no response format. The total score could range from 0 to 10 with higher scores corresponding to higher levels of general anxiety. The reliability of the scale was good in the present sample (polychoric Cronbach’s α = 0.72).

#### Visuo-spatial working memory

We administrated a computerized adaptation of the *Visual Pattern Test–Active Version* included in the standardized Italian BVS-Corsi battery (Mammarella et al., 2008). This task tests the ability to simultaneously retain and actively manipulate visuo-spatial information (Giofrè et al., 2013). The measure included twenty-one matrices of increasing size (from 4 to 16 squares) presented on a computer screen. Matrices were composed of white and coloured cells (the smallest had 4 total squares and 2 coloured cells, the largest had 16 total squares and 8 coloured cells). Each matrix was presented to the participants for 3 s, then it disappeared. Children were asked to mark on a blank matrix of the same size the location of the coloured cells moved by one row below (e.g., if the coloured cell was presented in the first row of the matrix, children had to mark the cell in the second row). Therefore, children were required to mentally shift the presented pattern one line below. The last row in the presentation matrix was always empty. Three matrices were presented for each level (span) of complexity, and a span was considered correct when a participant was able to correctly reproduce at least two of the three matrices for a given level. The final score was the maximum span level obtained (range from 2 to 8). Before administering the task, participants were given three practice trials with feedback. Task reliability is very good (Cronbach’s α = 0.90).

#### Math achievement

In order to evaluate students’ math abilities, we considered two different mathematical sub-domains: arithmetic skills and word problem-solving. Arithmetic skills, which encompass calculation ability as well as the understanding of mathematical symbols and the meaning of mathematical operations (Bisanz et al., 2005), were measured using three paper-pencil subtests drawn from the standardized Italian battery *ACMT 3 6–14* (Cornoldi et al., 2020): Math fluency, Inferences and Number matrices. The Math fluency subtest evaluates students’ ability to calculate basic arithmetic problems quickly and accurately. It requires solving 15 arithmetic operations (additions for third graders; additions and subtractions for fourth graders; additions, subtractions and multiplications for fifth graders) as quickly as possible in one minute. The Inference subtest assesses students’ arithmetic reasoning skills and comprises 12 items divided into three different types of tasks that must be completed in two minutes: in the first task participants had to solve 4 arithmetic operations involving images, in the second task they were asked to complete 4 operations by inserting the corresponding missing sign (+, -, ×, :), and in the third task they had to solve 4 operations by using the result of another similar operation as a cue. The Number matrices subtest assesses children’s arithmetic skills and math reasoning using numerical series. The participants had two minutes to solve 12 incomplete numerical matrices with the correct number. Responses were awarded a score of 0 or 1, depending on whether they were incorrect or correct, respectively. The final score consisted of the sum of all correct responses and could range from 0 to 39. Cronbach alpha in the present sample was 0.84.

To assess children’s word problem-solving skill, we administrated a task (adaptation from Hegarty et al., 1992; see Doz et al., 2023) consisting of 8 word problems, which required one or two elementary arithmetic calculations (addition and subtraction) to be solved. All the word problems included a relational term, such as *more than* or *less than*, and were similar to those assigned by teachers to students at the considered school grade. An example of word problem presented to third graders is: “At Bang-Bang, a teddy bear costs €18. This is €3 more than the same teddy bear on Aliexpress. How much does the teddy bear cost on Aliexpress?”. The order of the problems was randomized. The score of 0 or 1 was attributed to each problem according to whether the answer was wrong or correct, respectively. Its measurement range was between 0 and 8. Cronbach’s alpha in the present study was 0.82.

### Procedure

Participants were tested in three sessions administrated at school. Depending on school schedule and children’s availability, the sessions were separated by 7 to 10 days. The first session included the evaluation of students’ ER, MA and general anxiety. In the second session, participants were tested on visuo-spatial WM. In the final session, arithmetic skills and word problem-solving ability were assessed. Each session lasted approximately 30 to 40 min. Data were collected by trained researchers.

## Results

Statistical analyses were performed with SPSS software, version 28. Descriptive statistics (means and standard deviations) and correlations for all variables are reported in Table 1. It can be noted that both math achievement measures (arithmetic task and word problem-solving task) were significantly and positively correlated with ER and visuo-spatial WM task, but negatively with MA. Noteworthy, ER was statistically significantly and negatively correlated with MA and general anxiety. A statistically significant negative correlation emerged between MA and visuo-spatial WM.

**Table 1:** Descriptive statistics and bivariate correlations between all variables

Measure	M	SD	1.	2.	3.	4.	5.	6.	7.
1. Ego-resiliency	42.40	6.30	—						
2. Math anxiety	21.86	6.83	−0.42**	—					
3. General anxiety	3.51	2.19	−0.22**	0.39**	—				
4. Visuo-spatial WM	4.63	1.83	0.10	−0.23**	−0.03	—			
5. Arithmetic skills	17.76	5.75	0.16*	−0.33**	−0.14	0.33**	—		
6. Word problem-solving	2.72	1.33	0.17*	−0.22**	−0.12	0.28**	0.35**	—	
7. Age	9.47	0.88	0.02	−0.10	0.14	0.24**	0.15*	−0.09	—

Note. WM = working memory

* *p* < .05, ** *p* < .01

Since the correlations between the variables of interest resulted statistically significant, we proceeded by testing the hypothesized serial multi-mediational model. The Model 6 of the SPSS PROCESS macro was utilized (Hayes, 2017). 5000 bootstrap samples were employed to generate 95% bias-corrected and accelerated confidence intervals; if the 95% confidence interval of the indirect effect excludes zero, significance of the effect can be assumed (Hayes, 2017).

**Fig. 2:**
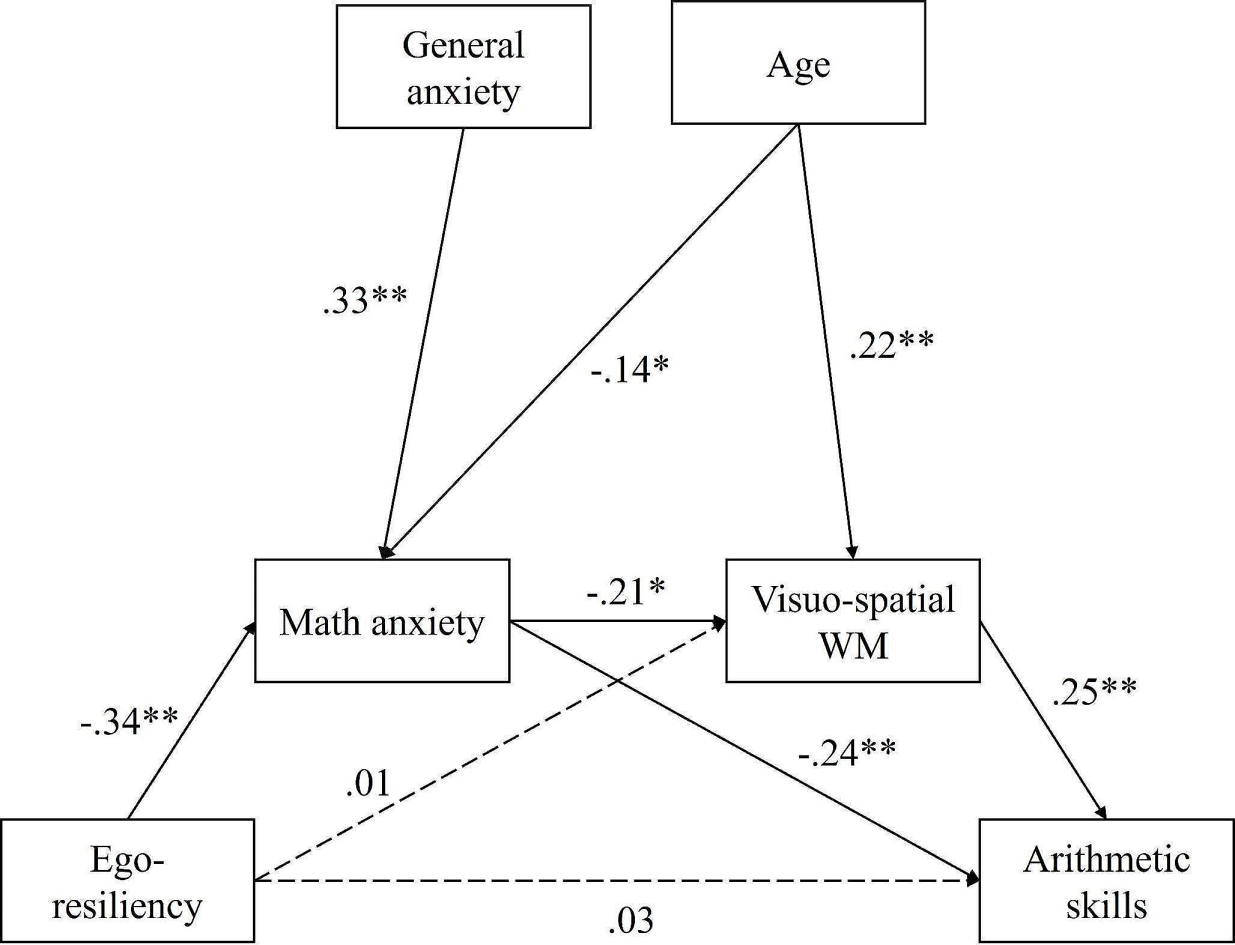
Results of the serial multi-mediation analysis with arithmetic skills as the outcome variable Note: R² = 0.188 Ego-resiliency was inserted as the focal regressor, math anxiety as the first mediator, visuo-spatial WM as the second mediator and arithmetic skills as the outcome variable. General anxiety and age were inserted as covariates. The figure shows the standardized regression coefficients, *β.* The dashed lines represent non-significant coefficients. WM = working memory * *p* < .05, ** *p* < .01

First, a serial mediation model was calculated placing ER as the focal regressor, arithmetic skills as the dependent variable, MA as the first serial mediator and visuo-spatial WM as the second mediator. General anxiety and age were inserted as covariates. The results of the multiple-mediation model are presented in Fig. 2. Findings revealed that ER was statistically significantly and negatively related with MA (*β* = –0.343, s.e. = 0.069, *p* < .001), but was not associated with visuo-spatial WM (*β* = 0.013, s.e. = 0.022, *p* = .861). In the second step, findings showed that MA was statistically significantly and negatively associated with visuo-spatial WM (*β* = –0.212, s.e.= 0.022, *p* = .011). Finally, MA was negatively associated with arithmetic skills (*β* = –0.238, s.e. = 0.068, *p* = .003), whereas visuo-spatial WM was positively related to arithmetic skills (*β* = 0.252, s.e. = 0.223, *p* < .001).

To estimate confidence intervals of the indirect effects, we used bias-corrected bootstrapping procedure. Using 5000 bootstrapped samples, two statistically significant indirect effects of ER on arithmetic performance were observed (see Table 2): (1) through MA (IE = 0.082, bootstrap s.e. = 0.033, 95% bias-corrected CI [0.022, 0.154]) and (2) through MA and visuo-spatial WM (IE = 0.018, bootstrap s.e. = 0.009, 95% bias-corrected CI [0.003, 0.041]). In other words, children’s ER impacted MA, which in turn had both a direct and indirect effect through visuo-spatial WM on arithmetic skills.

**Table 2:** Direct and indirect effects of ego-resiliency on arithmetic skills

Path	Standardized effect	Bootstrap s.e.	95% bias-corrected CI	
			Lower limit	Upper limit
Direct effect ER → arithmetic skills	0.030	0.067	–0.104	0.164
Indirect effects ER → MA → arithmetic skills	**0.082**	**0.033**	**0.022**	**0.154**
ER → visuo-spatial WM → arithmetic skills	0.003	0.021	–0.039	0.046
ER → MA → visuo-spatial WM → arithmetic skills	**0.018**	**0.009**	**0.003**	**0.041**

Note. ER = ego-resiliency; MA = math anxiety; WM = working memory; CI = confidence interval. Statistically significant effects are shown in bold

**Fig. 3:**
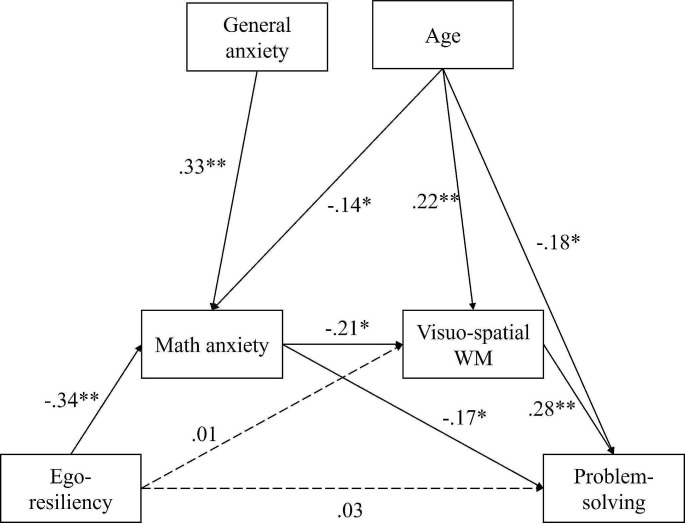
Results of the serial multi-mediation analysis with word problem-solving achievement as the outcome variable Note: R² = 0.135 Ego-resiliency was inserted as the focal regressor, math anxiety as the first mediator, visuo-spatial WM as second mediators and word problem-solving achievement as the outcome variable. General anxiety and age were inserted as covariates. The figure shows the standardized regression coefficients, *β.* The dashed lines represent non-significant coefficients. WM = working memory * *p* < .05, ** *p* < .01

Then, a second serial multiple-mediation model was calculated placing ER as the focal regressor, problem-solving accuracy as the dependent variable, MA as the first mediator and visuo-spatial WM as second mediator. The results of the second multiple-mediation model are presented in Fig. 3. Findings revealed that ER was statistically significantly and negatively related with MA (*β* = –0.343, s.e. = 0.069, *p* < .001), but was not associated with visuo-spatial WM (*β* = 0.013, s.e. = 0.022, *p* = .861). Then, MA was significantly and negatively associated with visuo-spatial WM (*β* = –0.212, s.e.= 0.022, *p* = .011). Moreover, MA was negatively associated with problem solving performance (*β* = –0.173, s.e. = 0.028, *p* = .040), and visuo-spatial WM (*β* = 0.283, s.e. = 0.093, *p* < .001) was positively associated with problem-solving performance. ER was not significantly associated with problem-solving outcome (*β* = –0.032, s.e. = 0.028, *p* = .680).

Using 5000 bootstrapped samples, two statistically significant indirect effects of ER on word problem-solving performance were observed (see Table 3): (1) through MA (IE = 0.022, bootstrap s.e. = 0.011, 95% bias corrected CI [0.001, 0.046]) and (2) through MA and visuo-spatial WM (IE = 0.006, bootstrap s.e. = 0.004, 95% bias-corrected CI [0.001, 0.015]). To put differently, children’s ER impacted their MA levels, which in turn have both a direct and indirect effect through visuo-spatial WM on problem-solving performance. In summary, the data show similar results for arithmetic skills and word problem-solving.

**Table 3:** Direct and indirect effects of ego-resiliency on word problem-solving performance

Path	Standardized effect	Bootstrap s.e.	95% bias-corrected CI	
			Lower limit	Upper limit
Direct effect ER → problem-solving	–0.017	0.028	–0.067	0.044
Indirect effects ER → MA → problem-solving	**0.059**	**0.030**	**0.002**	**0.121**
ER → visuo-spatial WM → problem-solving	0.003	0.023	–0.045	0.048
ER → MA → visuo-spatial WM → problem-solving	**0.020**	**0.010**	**0.003**	**0.046**

Note. ER = ego-resiliency; MA = math anxiety; WM = working memory; CI = confidence interval. Statistically significant effects are shown in bold

## Discussion

Much of the previous research has examined the relation between temperamental, emotional, and cognitive factors and math achievement in isolation (e.g., Alessandri et al., 2017; Kwok et al., 2007; Zheng et al., 2011). With this work we aimed to advance knowledge of how multiple factors – ER, MA and WM – can interact and determine math performance among primary school children. Thus, the primary purpose of this study was to provide a comprehensive model of the nature of the links between ER, MA, WM and math performance. In particular, we were interested in verifying a theoretically-based serial multi-mediational model (Block & Block, 1980; Eysenck & Calvo, 1992) in which we hypothesized that the relationship between ER and math performance would be serially mediated by MA (first mediator) and WM (second mediator). Secondly, we sought to better comprehend whether the tested factors could differently contribute to explain individual differences in different types of math skills (i.e., arithmetic skills and word problem-solving ability). Moreover, the current study advances previous studies by controlling for general anxiety and participants’ age, which are important factors to consider in children’s math learning (Donolato et al., 2020).

Our analysis revealed three main findings: (1) ER indirectly impacted math achievement through MA, and MA and visuo-spatial WM; (2) we replicated previous findings showing that visuo-spatial WM partially mediated the relationship between MA and math performance; (3), we found similar results also by considering two different math skills.

### The effect of ER is mediated by MA and WM

The results from this study clarify the role of ER in math achievement, one topic that has not been adequately addressed by previous empirical works. Firstly, in accordance with existing literature (Alessandri et al., 2017; Donolato et al., 2019, 2020; Kwok et al., 2007; Putwain et al., 2013), we found that children’s ER was positively correlated with math achievement, assessed with an arithmetic task and a word problem-solving task. This finding is consistent with the argument that being resilient, and thus being able to flexibly adapt when challenged or under pressure, supports school success in mathematics (Lee & Johnston-Wilder, 2017).

Secondly, serial multi-mediational analyses revealed that the relationship between ER and math achievement was indirect. Consistent with our hypothesis, ER was negatively associated with MA, which, in turn, negatively impacted math performance both directly and indirectly through visuo-spatial WM. This finding aligns with previous research demonstrating a negative relationship between ER and negative emotions (Chuang et al., 2006; Donolato et al., 2019, 2020; Putwain et al., 2013), and suggesting ER as a personal protective factor in anxiety development (Mammarella et al., 2018). Our study emphasizes the importance of ER specifically within the context of math anxiety. The data suggest that the trait of ER may facilitate individuals in adapting to situations when mathematics becomes difficult and challenging. In fact, although all children may experience difficulties while learning mathematics, higher ER levels can promote emotional regulation (Eisenberg & Morris, 2002) and perseverance (Borman & Overman, 2004), thus leading to reduced anxiety (Tellegen, 1985) and better academic performance (Putwain et al., 2013). On the other hand, lower levels of ER can be associated with diminished adaptability, difficulty responding to changing circumstances, a tendency to become disorganized under stress, poor recovery after distressing experiences (Eisenberg & Morris, 2002). These factors can contribute to perceive mathematics as a fearful subject and, over time, develop higher levels of MA. Therefore, our results support the theoretical model viewing ER as a construct closely related to individual’s ability to flexibly regulate emotions (Block & Block, 1980; Eisenberg et al., 2011).

It must be noted, however, that our results are partly in contrast with those found by Donolato et al. (2020). The authors assessed 5th to 8th graders on MA, general anxiety, test anxiety, ER and math achievement and found that ER was negatively associated with general anxiety, but not with test anxiety or MA. A possible explanation for these contrasting results could be referred to the participants’ age (Chuang et al., 2006; Gjerde et al., 1986). In fact, pre-adolescence represents a critical period when students start to engage with increasingly more demanding mathematical activities and experience increased general anxiety (Karande et al., 2018. Moreover, it should be noted that they are also more sensitive in evaluating their symptoms of anxiety compared to younger children. Therefore, future investigations should consider studying the ER-MA relationship in samples of different age or evaluating this relationship longitudinally.

### WM mediates the MA-math performance link

A second significant finding of the present study is that visuo-spatial WM partially mediated the MA-math performance link, for both math tasks, also after controlling for the influence of general anxiety and age. This finding is in accordance with previous studies among primary school children, indicating that MA negatively impacted students’ math achievement both directly and indirectly through visuo-spatial WM (e.g., Soltanlou et al., 2019; Živković et al., 2023). This aligns with the Processing Efficiency Theory (Eysenck & Calvo, 1992) and highlights that the interplay between emotional and cognitive factors may occur in school-aged children (Justicia-Galiano et al., 2017; Soltanlou et al., 2019; Szczygieł, 2021), mirroring patterns observed in older students and adults (Ashcraft & Kirk, 2001; De Caro et al., 2010; Ganley & Vasilyeva, 2014; Miller & Bichsel, 2004; Skagerlund et al., 2019). Specifically, children with higher levels of MA may experience more worries and intrusive thoughts, thereby occupying WM resources and limiting storage capacity for visual and spatial information, which is essential to solve a variety of math tasks. For example, in word problem-solving WM plays a fundamental role: word problems typically involve translating verbal information into mathematical representations, constructing a mental model of the problem situation, performing mental calculations, and generating appropriate problem-solving strategies (Hegarty et al., 1992; Mayer & Hegarty, 1996), and these mental abilities necessitates WM resources (Lee et al., 2009; Peng et al., 2016). Visuo-spatial WM, in particular, may facilitate the visualization and mental manipulation of visual representations in word problems. We speculate that this could be especially important for the word problems presented in the current study, which require a comparison of numerical quantities (Lewis, 1989). In this scenario, participants may have created a mental representation of the problem by spatially comparing the quantities. Additionally, visuo-spatial WM supports also arithmetic reasoning and arithmetic calculation (Kyttälä & Lehto, 2008).

It is important to note that MA also exhibited a direct negative relationship with math performance, suggesting the presence of additional mechanisms through which MA hinders students’ achievements. Recent studies posit that MA may influence students’ self-regulated learning and avoidance behaviours. For instance, research suggests that students with high MA perceive mathematical tasks as more challenging (Doz et al., 2023) and exhibit reduced level of perseverance when faced with mathematical difficulties (Gabriel et al., 2020; Yu et al., 2021), which may negatively influence students’ effort and overall performance.

### Different math tasks

Lastly, the study sought to better comprehend whether temperamental, emotional, and cognitive factors would play the same role in two different types of math skills (i.e., arithmetic skills and word problem solving ability). Our findings indicated a consistent pattern across both math skills. This is, ER was indirectly related to both arithmetic skills and word problem-solving performance through MA and MA and visuo-spatial WM. However, it can be noted that MA exhibited a stronger overall negative correlation (*r* = −.33) and a stronger direct relationship (*β* = −0.24) with the arithmetic task compared to the word problem-solving task (*r* = −.22; *β* = −0.17). One possible explanation for this disparity could be attributed to the inherent complexity of word problem-solving (Duque de Blas et al., 2021; Passolunghi et al., 2022), a task that heavily relies on WM (Fuchs et al., 2020; Peng et al., 2016) and as such most of the negative effect of MA may passes through its interplay with WM. Further research could explore this hypothesis by manipulating the complexity of the math tasks.

It is also important to note that the explained variance by the variables included in our models varies between different types of math skills. Specifically, the model explains a higher percentage of variance in arithmetic skills (19%) compared to word problem-solving (13.5%), suggesting that additional variables may be relevant especially in accounting for word problem-solving performance (see Fuchs et al., 2017; Lin, 2021). One such variable, not included in the current study, is verbal WM (Pina et al., 2014). Previous research has extensively shown that solving word problems relies not only on visuo-spatial WM but also significantly on verbal WM, as the task requires the maintenance and manipulation of words (Fuchs et al., 2020; Fung & Swanson, 2017; Peng et al., 2016; Zheng et al., 2011). Verbal WM is crucial for reading and text comprehension, extracting relevant information, organizing it meaningfully, and planning the steps to the solution (Lee et al., 2004). A second crucial variable, that could explain the difference in the explained variance, is language ability and text comprehension (Boonen et al., 2014; Cartwright et al., 2022; Fuchs et al., 2015; Passolunghi et al., 2022). In their longitudinal study, Fuchs et al. (2017) found that start-of-year language skills predicted second graders’ year-end word problem-solving ability more strongly than calculation ability, while initial arithmetic skills predicted year-end calculations more strongly than word problem-solving, suggesting that word problems and calculations may represent “distinct domains of academic performance” (p. 10). In accordance with this finding, our study highlights the importance of investigating specific math abilities separately, as each may exhibit unique characteristics and may involve different processes.

### Implications

Taken together, our study suggests that temperamental, emotional, and cognitive factors interact in determining children’s performance. Particularly, the results highlight the value of ER as a predictor of lower MA and, consequently, higher math performance, underlining the important and non-negligible role that personal assets have in math learning. These findings have significant implications in the educational setting, especially when developing school interventions aimed at enhancing math abilities and reducing negative attitude toward math in primary education. Although most of the resilience programs introduced in schools had the scope to enhance students’ mental health (e.g., Brunwasser et al., 2009), the results of this study indicate that interventions on ER might also have practical benefits for coping with specific negative emotions toward mathematics. Therefore, in addition to the traditional exercises used to reduce MA (e.g., Passolunghi et al., 2020; Samuel & Warner, 2021), novel MA interventions could involve activities related to the enhancement of ER. Such school-based programs can be designed to include various activities and metacognitive reflections that promote perseverance, effort and strategy beliefs, incorporate diagnostic feedback, instruct on how to seek assistance and support in the pursuit of mathematical learning, and encourage students to view mistakes as opportunities for improvement rather than failures (Lee & Johnston-Wilder, 2017). They can also focus on fostering classroom collaboration and help students define success and competence in relation to personal objectives and values instead of competing with others (Martin & Marsh, 2006). Finally, such interventions can be run in parallel with more specific math trainings, which are generally targeted on improving math skills and their underlying cognitive processes. Indeed, the latter might be ineffective if children do not learn how to handle their negative emotional states or are not enough resilient.

### Limitations and future directions

Some limitations should be taken into account when interpreting the present results. Firstly and most importantly, the serial multi-mediation analysis relies on correlational procedures and, as such, causal relations should not be inferred. Our study provided some evidence on the association between temperamental, affective and cognitive factors, and math abilities, however longitudinal studies are needed to accurately determine the direction and temporal relation of the links between the different variables involved. For instance, recent studies suggest that the relationship between MA and math performance is reciprocal: Pekrun et al. (2023) investigated within-person relations of students’ achievement and emotions in mathematics over 5 school years and found that achievement negatively predicted negative emotions, and these emotions, in turn, were negative predictors of math achievement. Similarly, as pointed out by Putwain et al. (2013), it is likely that also the constructs of ER and MA would interplay reciprocally over time, so future research may consider employing a longitudinal approach to establish evidence for the causal relationship between ER and MA. Another limitation of the current work is its reliance on individual tasks, which − although recognized as reliable − may not capture the full complexity of the constructs under investigation. Future research could benefit from utilizing more tasks and structural equation modeling to provide a more comprehensive analysis. Finally, although we investigated the effects of general anxiety, we did not control for children’s test anxiety, which has been found to correlate with ER (Putwain et al., 2013), MA and math achievement (Caviola et al., 2022). Therefore, future studies could consider this factor as well.

## Conclusions

Given a growing trend of “mathematization of society” (Jablonka & Gellert, 2007), the world necessitates more than ever to equip students with the necessary mathematical knowledge and skills to effectively participate, as aware citizen, in this “mathematized world”. The present study highlights the intricate interplay of multiple factors in predicting math achievement. Specifically, the paper underlines the role of ER as a significant individual difference variable that influences math performance through its impact on MA, which subsequently affects visuo-spatial WM resources. Recognizing the protective role of ER, on one hand, enables us to broaden our comprehension of students’ diverse responses to math learning and, on the other hand, provides insights for developing more effective interventions to contrast negative feelings (Passolunghi et al., 2020).

## Data Availability

All data and research material are available by emailing the corresponding author.
